# A bioactive flavonoid from *Pavetta crassipes *K. Schum

**DOI:** 10.1186/2191-2858-1-14

**Published:** 2011-10-04

**Authors:** Isaac A Bello, George I Ndukwe, Oladimeji T Audu, James D Habila

**Affiliations:** 1 Department of Chemistry, Ahmadu Bello University, Zaria, Nigeria

**Keywords:** bio-activity, rutin, *Pavetta crassipes*, antimicrobial, phytochemistry, structure elucidation

## Abstract

**Background:**

In our continued search for bioactive compounds from plants, conscious effort is being made to rapidly analyze ethnobotanical plants used for treating various ailments by traditional healers before this information is irrevocably lost as societies advance and rural communities become urbanized.

**Results:**

A compound isolated from the aqueous extract of *Pavetta crassipes *leaves showed activity against some pathogenic microorganisms which included *Streptococcus pyogenes, Corynebacterium ulcerans, Klebsiella pneumoniae, Neisseria gonorrhoeae, Pseudomonas aeruginosa*, and *Escherichia coli *at a concentration < 50 mg/mL. The compound had minimum inhibitory concentration ranging from 6.25 to 12.5 mg/mL and minimum bactericidal concentration ranging from 12.5 to 25 mg/mL. The compound was identified using 1D and 2D NMR experiments and comparison with literature data as quercetin-3-*O*-rutinoside.

**Conclusions:**

This has supported the ethnomedicinal use of the plant, confirmed its activity, and has also provided an easy and simple method for isolating this compound which has a lot of pharmaceutical and cosmetic applications from a new source.

## Background

Plants have a long history of use all over the world for the treatment of different diseases and complaints. In certain African countries, up to 90% of the population still relies exclusively on plants as a source of medicines and many of these plants have been documented [1]. The available knowledge on the use of plant preparations in traditional medicine is enormous but if this is not rapidly researched, indications as to the usefulness of this vegetable treasure-house will be lost with succeeding generations [1].

Africa is reputed for the extraordinary richness of its flora, totalling several tens of thousands of species. Environmental degradation provides a threat to biological diversity, but the sub-Saharan region still boasts of a wide variety of indigenous species. Based on careful observation and a judicious choice of plants, it is possible to discover interesting new natural products [2].

*Pavetta crassipes *K. Schum. (Rubiaceae) is a low shrub of the savannah. In Nigeria, the leaves of this plant are used medicinally in the management of respiratory infections and abdominal disorders. The leaves are also used in Tanzania in the treatment of gonorrhoeae. In Central Africa, the acid infusion of the leaves is taken as a cough remedy [3]. The leaves are eaten by some native tribes pounded up with other food, or boiled in the slightly fermented water in which cereals have been left to steep, and mixed with pap. The sap is a coagulant of rubber latex [4].

Alkaloid extracts from the plants have been shown to have significant anti-malarial activity [5]. The ethanol extract has been shown to lower the blood pressures of cats and rats in a dose-dependent manner [6].

In view of these medicinal uses, *P. crassipes *is a good candidate for screening for bioactive compounds. It is imperative that a study of the plant be carried out with a view to justifying the claims by the traditional users and possibly isolating and characterizing the compound(s) responsible for the perceived activity. We now report the isolation and characterization of a bioactive compound from the leaves of *P. crassipes *and its antimicrobial properties.

## Results

### Phytochemical screening

The phytochemical studies revealed the presence of flavonoids in the leaves of the plant. Extraction of the leaves led to the isolation of a flavonoid glycoside.

### Antimicrobial screening

The results of the antimicrobial studies showed that the compound had a remarkable activity at 50 mg/mL against six of the ten microorganisms tested.

### Spectroscopy

The compound was analyzed using^1^H NMR,^13^C NMR, DEPT, COSY, NOESY, HMBC, and HSQC experiments. Comparison of the results with literature data [7-11] confirmed the compound as quercetin-3-*O*-rutinoside.

## Discussion

Flavonoids are widely distributed in plants. They are known to be responsible for the yellow or red/blue pigmentations in flowers and also provide protection from attack by microorganisms and insects. The widespread distribution of flavonoids, their variety, and their relatively low toxicity compared to other active plant metabolites (for instance alkaloids) had led to many animals, including humans, ingesting significant quantities in their diet without problems. Flavonoids have been referred to as "nature's biological response modifiers" because of the strong experimental evidence of their inherent ability to modify the body's reaction to allergens, viruses, and carcinogens. They show anti-allergic, anti-inflammatory, anti-microbial, and anti-cancer activity [12].

Antimicrobial studies showed that the plant had zones of inhibition ranging from 15 to 22 mm. It however could not inhibit the growth of *S. aureus, B. subtilis, S. typhii *and *C. albicans*. The zones of inhibition showed that the compound had remarkable activity when compared to standard drugs [13].

MIC and MBC studies showed that the compound inhibited the growths of *Streptococcus pyogenes*, *Klebsiella pneumoniae*, and *Neisseria gonorrhoeae *at a concentration of 12.5 mg/mL with an MBC at 25 mg/mL. *Corynebacterium ulcerans*, *Escherichia coli*, and *Pseudomonas aeruginosa *were all inhibited at a concentration of 6.25 mg/mL with corresponding MBC at 12.5 mg/mL (Table 1).

**Table 1:** Summary of MIC and MBC of the compound (mg/mL)

Organisms	MIC	MBC
*E. coli*	6.25	12.5
*P. aeruginosa*	6.25	12.5
*S. pyogenes*	12.5	25.0
*C. ulcerans*	6.25	12.5
*K. pneumoniae*	12.5	25.0
*N. gonorrhoeae*	12.5	25.0

**Figure 1:**
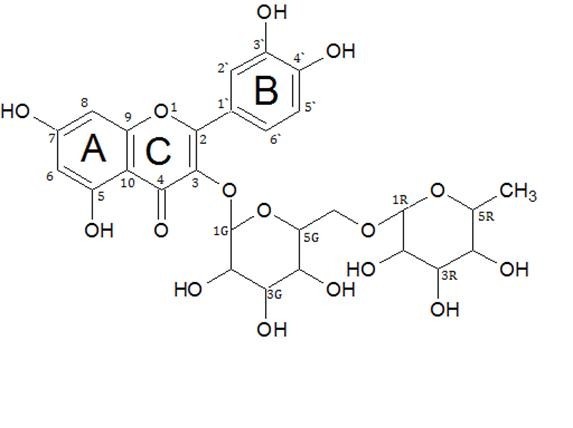
**Quercetin-3-*O*-rutinoside**. Structure of the isolated compound.

The^1^H NMR spectrum summarized in Table 2 shows the following signals in the aromatic region with patterns similar to those of flavonoids [14]. Doublets at δ 6.19 (*J *= 1.88 Hz), 6.41 (*J *= 1.8 Hz), 7.53 (*J *= 8.08 Hz), 7.55 (*J *= 7.56 Hz) 6.85 (*J *= 7.84 Hz), and a singlet at 12.62 which corresponds to protons attached to the carbon atoms at positions C-6, C-8, C-2', C-6', C-5', and the -OH at C-5, respectively (Figure 1). The signal at δ 0.97 (*J *= 6.12 Hz) corresponds to the signal expected from the methyl group of a rhamnose moiety. The signal at δ 5.32 (*J *= 7.44 Hz) indicates that the anomeric glucose proton was in the beta configuration, while the signal at δ: 4.37 (*J *= 7.6 Hz) indicates that the anomeric rhamnose proton is in the alpha configuration [15]. The signals between δ 3.00 and 4.00 belong to the other protons of the sugar moiety.

**Table 2:** ^13^C and ^1^H chemical shifts assignments for the compound

Position	^13^C (400 MHz, DMSO-d_6_)	^1^H (400 MHz, DMSO-d_6_)
2	156.4	
3	133.2	
4	177.3	
5	156.6	12.62 (1H, s, 5-OH)
6	98.6	6.19 (1H, d, J = 1.88)
7	164.0	
8	93.6	6.41 (1H, d, *J *= 1.80)
9	161.1	
10	103.9	
1'	121.1	
2'	115.2	7.53 (1H, d, *J *= 8.08)
3'	144.6	
4'	148.3	
5'	116.2	6.85 (1H, d, *J *= 7.84)
6'	121.6	7.55 (1H, d, *J *= 7.56)
1^G^	101.1	5.32 (1H, d, *J *= 7.44)
2^G^	73.9	3.08 (1H, d, *J *= 9.28)
3^G^	75.8	3.23 (1H, d, *J *= 6)
4^G^	69.9	3.26-3.36 (3H)
5^G^	76.3	3.21 (1H, d, *J *= 5.52)
6^G^	66.9	3.26-3.36 (3H)
1^R^	100.7	4.37 (1H, d, *J *= 7.6)
2^R^	70.3	3.04 (1H, d, *J *= 2.68)
3^R^	70.5	3.69 (1H, d, *J *= 10.4)
4^R^	71.8	3.26-3.36 (3H)
5^R^	68.2	3.39 (1H, d, *J *= 1.76)
6^R^	17.6	0.97 (3H, d, *J *= 6.12)

The^13^C NMR spectrum summarized in Table 2 indicated a total of 27 carbon atoms. Fifteen of which were methine (CH) carbon atoms, one was a methyl (CH_3_) carbon atom, one was a methylene (CH_2_) carbon, and ten were quaternary (C) carbon atoms confirmed from the DEPT 90 and DEPT 135 experiments.

The methine (CH) signals at δ 98.6 and 93.6 belong to the A-ring (Figure 1) at positions 6 and 8, respectively, while the signals at 116.2, 115.2, and 121.6 belong to the B-ring (Figure 1) at positions 2', 5', and 6', respectively, and the signals at 101.1, 73.9, 75.8, 69.9, 76.3, 100.7, 70.3, 70.5, 71.8, and 68.2 are located on the disaccharide moiety. The methyl (CH_3_) signal at δ 17.6 was attributed to the terminal methyl group on the rhamnose unit at position 6. The methylene (CH_2_) signal at δ 66.9 was attributed to the CH_2 _carbon at position six of the glucose unit. The quaternary (C) carbon atoms at δ 156.4, 133.2, 177.3, 161.1, 164.0, 156.6, and 103.9 are on the A-ring while the signals at δ 121.1, 144.6, and 148.3 are located on the B-ring. The signals at δ 101.1, 73.9, 75.8, 69.9, 76.3, 100.7, 70.3, 70.5, 71.8, 68.2, 66.9, and 17.6 are consistent with those of rutinosyl (Table 2).

These assignments were confirmed by the COSY, NOESY, HSQC, and HMBC experiments.

## Conclusions

The results from this research have supported the ethnomedicinal uses of this plant in the treatment of respiratory infections, abdominal disorders, gonorrhea, and as a cough remedy. These diseases can be caused by the respective microorganisms tested. The compound was purified by re-crystallization and characterized as quercetin-3*O*-rutinoside. Further studies are going on to establish other phytochemicals in the plant.

## Methods

### Extraction

The fresh plant (1 kg) was extracted using hot water and filtered. A yellow solid (13.5 g) was precipitated on standing for a few hours. It was filtered using a Buchner funnel and trap under vacuum and re-crystallized from redistilled methanol to yield yellow needle-like crystals (4.52 g).

### Phytochemical screening

Phytochemical analysis was carried out on the re-crystallized compound using the method set out by Brain and Turner [16] and Trease and Evans [17].

#### Shinoda's test for flavonoids

About 5 mg of the compound was dissolved in ethanol. 3 mg magnesium powder was then added followed by few drops of conc. HCl. An orange coloration indicated the presence of flavonoids.

#### Ferric chloride test for flavonoids

About 5 mg of the compound was dissolved in ethanol (2 mL). A few drops of 10% ferric chloride solution were added. A green-blue coloration indicated the presence of a phenolic hydroxyl group.

#### Sodium hydroxide test for flavonoids

About 5 mg of the compound was dissolved in water, warmed, and filtered; to this solution (2 mL), 10% aqueous sodium hydroxide was added. This produced a yellow coloration. A change in color from yellow to colorless on addition of dilute hydrochloric acid was an indication for the presence of flavonoids.

### Antimicrobial screening

The antimicrobial activity was determined using some pathogenic microorganisms. The microorganisms were obtained from the Department of Medical Microbiology, Ahmadu Bello University Teaching Hospital, Zaria, Nigeria. All isolates were checked for purity and maintained in slants of blood agar.

A solution of 0.5 g of the compound was made using 10 mL DMSO. This solution was used to check the antimicrobial activity of the compound. A control experiment was also set up using DMSO.

Blood agar base (Oxoid, England) was prepared according to the manufacturer's instructions. This was then sterilized at 121°C for 15 min using an autoclave and was allowed to cool. The sterilized medium (20 mL) was pipetted into sterilized Petri dishes, covered, and allowed to cool and solidify.

The Petri dishes containing the medium were seeded with the test organisms by the spread plate technique and were left to dry for half an hour.

Filter paper disks were cut and sterilized at 160°C for 30 min. The sterilized paper disks were then dropped into the solutions of the extracts and were dried at 45°C. The dried disks were then planted on the medium previously seeded with the test organisms. The plates were incubated at 37°C for 24 h after which they were inspected for the zones of inhibition of growth. The zones were measured and recorded in millimeters by the use of a pair of dividers and a ruler.

### Minimum inhibition concentration

Minimum inhibition concentration (MIC) of the compound was carried out on the microorganisms that were susceptible to it and was carried out using the broth dilution method as described by Bauer et al. [18]. Nutrient broth (Oxoid, England) was prepared according to the manufacturer's instructions. 10 mL each was dispensed into five sets of screw cap test tubes and sterilized at 121°C for 15 min. The test tubes were allowed to cool down.

McFarland's turbidity standard scale number 0.5 was prepared. 10 mL normal saline was used to make a turbid suspension of the microorganisms. Dilution of the microorganisms was done continuously in the normal saline until the turbidity matched that of the McFarland's scale by visual comparison. At this point, the microorganisms had a density of 3 × 10^8 ^cfu/mL.

Serial dilution of the compound was made using the nutrient broth and the following concentrations were obtained: 50, 25, 12.5, 6.25, and 3.125 mg/mL. Having obtained the different concentrations, 1 mL of the microorganism in the normal saline was inoculated into the different concentrations of the compound in the broth and was incubated at 37°C for 24 h. The lowest concentration that showed no turbidity (clear solution) was recorded as the MIC.

### Minimum bactericidal/fungicidal concentration

This was carried out to determine whether the microorganisms could be completely killed or their growth could only be inhibited.

Blood agar base (Oxoid, England) was prepared according to the manufacturer's instructions. The solution was sterilized at 121°C for 15 min using an autoclave and poured into sterilized Petri dishes. The contents of the MIC test tubes in the serial dilution were sub-cultured on the Petri dishes by dipping a sterile wire loop into each test tube and streaked on the surfaces of the Petri dishes. The Petri dishes were incubated at 37°C for 24 h after which they were observed for growth. The minimum bactericidal/fungicidal concentration (MBC/MFC) was the Petri dish with the lowest concentration of the compound that had no growth of the microorganisms.

## Competing interests

The authors declare that they have no competing interests.
